# Acute respiratory failure as a manifestation of an arachnoid cyst

**DOI:** 10.4103/0972-5229.40951

**Published:** 2008

**Authors:** Lalitha V. Pillai, Gopal Achari, Sanjay Desai, Vinayak Patil

**Affiliations:** **From:** Department of Critical Care, Lokmanya Hospital, Chinchwad, Pune - 411 033, Maharashatra, India

**Keywords:** Cystoperitoneal shunt, posterior fossa arachnoid cyst, respiratory arrest

## Abstract

Arachnoid cysts are the most common congenital cystic lesions in the brain occurring in the middle fossa, suprasellar region and occasionally in the posterior fossa. Conventionally all cysts are considered as benign and symptoms are attributed to expansion of cysts causing compression of adjacent neurological structures, bleeds within the cyst or due to the development of acute hydrocephalus. We are reporting this case of a 15-year-old female patient with non-progressive weakness in the limbs since the age of seven years who presented with acute onset syncopal attacks and respiratory failure. She was intubated and ventilated. An magnetic resonance imaging scan showed large posterior fossa cyst extending up to mid second cervical vertebra causing compression of the medulla and pons, with mild hydrocephalus. After a failed attempt to wean her from the ventilator a cysto peritoneal shunt surgery was performed following which she was weaned from the ventilator successfully. Weakness in the upper and lower limbs, which had increased in the preceding month, also improved following the surgery.

## Introduction

Arachnoid cysts form 1% of all intracranial lesions and are collections of cerebrospinal fluid within the arachnoidal lining of the brain probably present at birth or developing soon after.[1,2] Symptomatic arachnoid cysts present with seizures, mental retardation, cognitive function impairment, ataxia, unusual bobbing of the head in infants and progressive weakness of lower limbs in cases of spinal arachnoid cyst.[3–5] Chiari malformation, syringohydromyelia have also been associated with these cysts.[6–8] Suprasellar cysts may produce visual impairment, obstructive hydrocephalus and endocrinal dysfunction in the form of precocious puberty, amenorrhea, developmental delay and retarded skeletal growth.[3,4,9] Patients with posterior fossa cysts have reported vague symptoms or headache and gait disturbances.[7,8,10] Presentation with hearing loss, imbalance and tinnitus has been mistaken for Meniere's disease.[11] We are reporting a case of posterior fossa arachnoid cyst that presented with acute respiratory failure.

## Case Report

A 15-year-old female patient from a poor socioeconomic background was admitted at 10 pm with history of syncopial attacks followed by vomiting half an hour prior to admission. At seven years of age she developed weakness and wasting of upper and to a lesser extent of the lower limbs, which was preceded by headache and pain in the neck. This she attributed to a fall she had while playing and subsequently because of the weakness she was unable to continue schooling. There was no sensory disturbance, bowel or bladder involvement and she had never been investigated prior to the present admission. During the preceding month there was a rapid deterioration in the power with upper limbs being more affected than lower limbs and continuous pain in the neck. The weakness in the limbs increased to the extent that she was unable to hold objects or feed herself and could only sit in bed. A day preceding the admission she developed loose motions for which she was treated by a general practioner. Two hours prior to admission she had three episodes of syncope followed by difficulty in breathing.

On arrival in emergency, she was conscious, alert, febrile with a temperature of 100°F and cyanosed. She had a pulse rate of 120/min, BP 110/60 mm of Hg; RR 30/min. Respiration was shallow and rapid with active accessory muscles of respiration. Wasting of the muscles of upper limbs more than the lower limbs were noted. Her lab investigations were as follows, Hemoglobin 11.2 gm/dl, leukocyte count 8800/cumm, blood sugar 143 mg/dl, Na^+ −^ 131mEq/L, K^+−^3.9 mEq/L, Cl^−^ 94 mEq/L with normal coagulation profile, renal and liver function tests. ABG showed a pH - 7.12, PaCO_2_ - 66.1 mm Hg, PaO_2_ - 48.9 mm Hg, H_2_ CO_3_ -20.6 mmol/l bedside echocardiography X-ray chest and sonography were normal.

**Figure 1:**
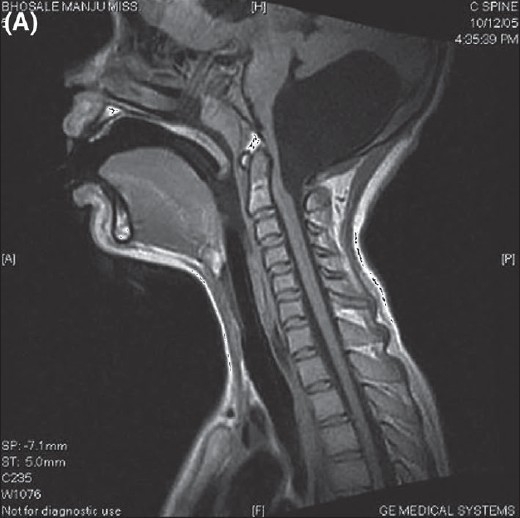

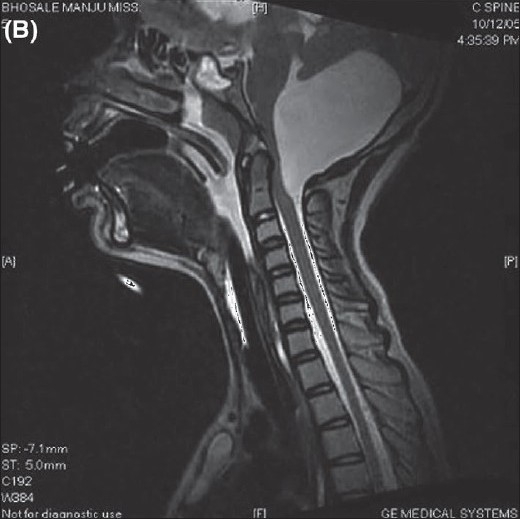
(A) MRI showing large posterior fossa archnoid cyst. (B) MRI showing posterior fossa archnoid cyst causing compression of adjacent structures

Before a detailed neurological examination could be performed the patient became drowsy and developed bradycardia. She was immediately intubated and taken on controlled ventilation. Her sensorium improved and she became hemodynamically stable. MRI scan showed a large posterior fossa arachnoid cyst in midline with a dimension of 3.2 cm × 4.7cm × 3.6 cm causing severe compression over the cerebellar vermis [Figures 1A and B]. It displaced the fourth ventricle antero-superiorly and caused mass effect over the dorsal aspect of pons and medulla, which appeared, compressed against the clivus. It also extended into the cervical spinal canal up to mid second cervical vertebral level. Mild obstructive hydrocephalus and periventricular ooze were noted.

**Figure 2:**
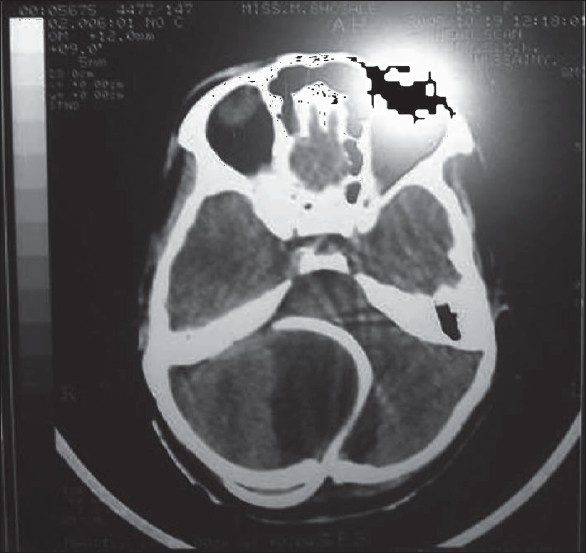
Post operative CT sacn with shunt *in situ*

She was ventilated on spontaneous mode for the next two days. As she was alert and asymptomatic she was extubated on the third day after a T-piece trial. Four hours later she had to reintubated and ventilated because of progressive respiratory distress with retention of CO_2_ and increasing drowsiness. Cysto peritoneal shunt was performed on the fourth day of admission. Fluid from the cyst was clear, colorless with 22 mg/dl protein, 47 mg/dl sugar. Two days later she was successfully weaned from the ventilator. A convulsion on the fifth postoperative day was treated with loading and maintenance dose of phenytoin. Repeat CT scan [Figure 2] showed the shunt tube *in situ* in posterior fossa, with regression in size of the cyst and hydrocephalous as compared to the previous study. The size now was 3.2 cm × 4.1 cm × 3.5 cms. Subsequent hospital stay was uneventful. She was discharged on the fourteenth day. At the time of discharge she was able to walk with support and hold objects in her hand. There was further improvement in the proximal and distal muscles in the subsequent visits but five months later she was lost to follow-up.

## Discussion

This case is being reported for the unusual presentation of a large posterior fossa arachnoid cyst with acute respiratory failure. Midline posterior fossa cysts account for 10% of all arachnoid cysts. The exact incidence of mortality and morbidity due to arachnoid cyst is not known as most of the cysts are found incidentally or have subtle symptoms inspite of the large size. Most arachnoid cysts have presented in infancy or in early childhood.[3,4,12] In Zain Alabedeen *et al* series of 25 patients, 67% of the patients presented at less than 15 years of age.[3] While our patient developed muscle weakness at seven years, unfortunately she was never investigated until the present admission at 15 years of age. Her dominant symptom was muscle weakness in the childhood. It is not uncommon to find coexistent syringomyelia with posterior fossa arachnoid cyst.[7,8] In our case there was no syrinx. There was an unusual extension of the cyst upto the second cervical spine, which may have contributed to the neurological symptoms.

Arachnoid cysts are usually said to remain stable in size, although there are reports of cysts disappearing or slowly growing in size.[3,4,12–16] The patient here, had two episodes of neurological deterioration, one at seven years of age and the other a month prior to the present admission. Neck pain and syncopal attacks preceded both the episodes and were probably the heralding symptoms of cyst enlargement. It is tempting to correlate the onset and deterioration in the patient's symptoms to the changes in the cyst size. Midline posterior fossa tumor presenting with episodes of unconsciousness has been reported in the presence of acute hydrocephalus.[7,8]

Increase in size of the cyst has been ascribed to secretion of fluid by the ependymal cells, fluid ingress due to an osmotic gradient or trapping of fluid by a ball-valve mechanism.[17] Other cause of neurological worsening have been due to rupture of the arachnoid cysts into the subdural space or because of intracystic hemorrhage.[18] Composition of cyst fluid is said to be similar to cerebral spinal fluid. Elevated protein levels are hypothesized to cause expansion of the cyst.[19] In our case the fluid protein content was not raised nor was there a rupture or bleed within the cyst, hence other mechanisms must have played a role in increasing the size of the cyst.

Since the cyst size usually remains stable it was as felt that the intercurrent infection had contributed to the sudden respiratory failure in our patient. Hence weaning from the ventilator was attempted. When weaning failed shunt surgery was decided on in an attempt to reduce the pressure as most symptoms in patients with arachnoid cysts have been due to the pressure or compression of the neurological structures by the cyst. The follow-up CT scan showed a reduction in the size of the cyst to corroborate our clinical findings. As to the incident of postoperative seizures, in Zain Alabedeen *et al* series too there was an incident of postoperative seizure in a patient who underwent craniotomy and seizures have been the presenting symptom in middle fossa cysts.[3]

Surgery for arachnoid cysts has resulted in reduction of headaches, improvement in cognitive function and power of the limbs.[3,4,5,7,8,20,21] In our case marked improvement in her ability to breathe independent of the machine followed surgery. Further improvement in her clinical status on follow-up visit justified our decision to operate. Acute respiratory failure in this patient who appeared to have a chronic progressive neurological disease was reversible following cysto peritoneal surgery.

## Conclusion

The acute enlargement of the posterior fossa arachnoid cyst produced compression of the cervicomedulary junction resulting in acute respiratory failure. The syncopal attacks could have been due to acute hydrocephalus. Persistent pain in the neck may have been a prodromal symptom of cyst expansion Intercurrent infection and electrolyte imbalance probably contributed to immediate deterioration but it alone could not explain the respiratory failure because she could be weaned of the ventilator only after the cysto peritoneal shunt, following which there was improvement in the limb power. The reduction in cord compression has been documented in postoperative scan.

## References

[1] Robinson RG (1971). Congenital cysts of the brain: Arachnoid malformations. Neurol Surg.

[2] Flodmark O (1992). Neuroradiology of selected disorders of the meninges, calvarium and venous sinuses. AJNR Am J Neuroradiol.

[3] Jamjoom ZA (1997). Intracranial arachnoid cysts: Treatment alternatives and outcome in a series of 25 patients. Ann Saudi Med.

[4] Artico M, Cervoni L, Salvati M, Fiorenza F, Caruso R (1995). Supratentorial arachnoid cysts: Clinical and therapeutic remarks on 46 cases. Acta Neurochir (Wien).

[5] Williams PW, Bergh WM, Vandertop WP (2000). An arachnoid cyst presenting as an intramedullary tumour. J Neurol Neurosurg Psychiatry.

[6] Sleinman M, Assarker R, Bourgeois P, Lejeune JP, Soto-Ares G (2000). Suprasellar arachnoid cyst associated with syringomyelia. Neurochirurgie.

[7] Bauer AM, Mueller DM, Oró JJ (2005). Arachnoid cyst resulting in tonsillar herniation and syringomyelia in a patient with achondroplasia. Case report. Neurosurg Focus.

[8] Jain R, Sawlani V, Phadke R, Kumar R (2000). Retrocerebellar arachnoid cyst with syringomyelia: A case report. Neurol India.

[9] Brauner R, Pierre-Kahn A, Nemedy-Sandor E, Rappaport R, Hirsch JF (1987). Precocious puberty caused by a suprasellar arachnoid cyst. Analysis of 6 cases. Arch Fr Pediatr.

[10] Erdincler P, Kaynar MY, Bozkus H, Ciplak N (1999). Posterior fossa arachnoid cysts. Br J Neurosurg.

[11] O'Reilly RC, Hallinan EK (2003). Posterior fossa arachnoid cysts can mimic. Meniere's disease. Am J Otolaryngol.

[12] Harsh NG, Edwards MS, Wilson CB (1986). Intracranial arachnoid cysts in children. J Neurosurg.

[13] Mori T, Fujimoto M, Sakae K, Sakakibara T, Shin H, Yamaki T (1995). Disappearance of arachnoid cysts after head injury. Neurosurgery.

[14] Kumagai M, Sakai N, Yamada H, Shinoda J, Nakashima T, Iwama T (1986). Postnatal development and enlargement of primary middle cranial fossa arachnoid cyst recognized on repeat CT scans. Childs Nerv Syst.

[15] Rao G, Anderson RC, Feldstein NA, Brockmeyer DL (2005). Expansion of arachnoid cysts in children: Report of two cases and review of the literature. J Neurosurg.

[16] Becker T, Wagner M, Hofman E, Warmuth-Metz M, Nadjmi M (1991). Do arachnoid cysts grow? A retrospective CT volumetric study. Neuroradiology.

[17] Dyck P, Gruskin P (1997). Supratentorial arachnoid cysts in adults. A discussion of two cases from a pathophysiologic and surgical perspective. Arch Neurol.

[18] Ibarra R, Kesava PP (2000). Role of MR imaging in the diagnosis of complicated arachnoid cyst. Pediatr Radiol.

[19] Sandberg DI, McComb JG, Krieger MD (2005). Chemical analysis of fluid obtained from intracranial arachnoid cysts in pediatric patients. J Neurosurg.

[20] Wester K, Hugdhal K (1995). Arachnoid cysts of the left temporal fossa: impaired preoperative cognition and postoperative improvement. J Neurosurg Psychiatry.

[21] Pierre-Kahn A, Capelle L, Brauner R, Sainte-Rose C, Renier D, Rappaport R (1990). Presentation and management of suprasellar arachnoid cysts. Review of cases. J Neurosurg.

